# Prostate Cancer Patterns and Trends in the Eastern Cape Province of South Africa; 1998–2017

**DOI:** 10.3389/fpubh.2022.882586

**Published:** 2022-04-28

**Authors:** Thendo Michael Ramaliba, Nomfuneko Sithole, Akhona Ncinitwa, Nontuthuzelo I. M. Somdyala

**Affiliations:** Burden of Disease Research Unit, South African Medical Research Council, Cape Town, South Africa

**Keywords:** prostate cancer, incidence rate, rural population, Eastern Cape, magisterial area

## Abstract

**Background:**

Globally, prostate cancer is rated the second most common cancer and the sixth leading cause of death. In South Africa, it is ranked as leading cancer among men. This study describes prostate cancer patterns and trends in the rural Eastern Cape Province population.

**Methodology:**

Secondary data were used from which a sample of 723 prostate cancer (C61) patients was extracted from the database into STATA version 14.0 for descriptive analysis. A direct standardization method was used to estimate age-specific and age-standardized incidence rates. Keyfitz method was used to calculate the standard error and confidence interval, whereas the Joinpoint program the annual percentage change.

**Results:**

The mean age was 64 years, with a standard deviation of 9.9. Trends in prostate cancer incidence increased significantly (*p* = 0.026) from 7.4% in 2010 to 12.6% in 2017. Incidence rates varied across the region, with the lowest of 4.5 per 100,000 in 1998 to the highest of 21.4 per 100,000 in 2017 period. Lusikisiki had the highest incidence rates of 53.4 per 100,000 population (95% CI 0.8–61.4), while Centane with 21.7 per 100,000 (95% CI 2.3–27.6) rated the second. Other magisterial areas showed a constant increase (*p* > 0.05) throughout the observation period except for Idutywa and Willowvale, with no apparent increase. Conversely, in Butterworth, incidence rates decreased from 15.2 per 100 000 (95% CI 8.6–21.9) to 11.5 per 100,000 (95% CI 6.2–16.7).

**Conclusion:**

As experienced globally and regionally, prostate cancer has become a public health concern in this population. Incidence variations across the surveillance area in the Eastern Cape were noted with hotspots.

## Introduction

Globally, prostate cancer remains the most frequently diagnosed cancer among men and is the fifth leading cause of death in 2020 (1). There was an estimated incidence rate of 37.5 per 100 000 and 1.4 million new cases worldwide (1). Prostate cancer incidence rates varied by 189-fold across 186 countries, with age-standardized incidence rates (ASIR) ranging widely from 1.0 per 100,000 in Bhutan to as high as 189.1 in Guadeloupe in 2018 (2). In recent research, Europe (Lithuania) had the highest incidence rates of 128.9 per 100,000, followed by Central and Southern America with 122.3 per 100,000 (2). Oceania had the third highest with 119.4 per 100,000 (3).

Evidence suggests a rise in prostate cancer incidence rates in low-middle-income countries (LMICs), including Africa (4, 5). Improved life expectancy and adopted westernized lifestyle are risk factors associated with the increased prostate incidence rates (6). There are also suggestions that these increased rates could be related to variations of screening and testing patterns across the Africa region (7). New cases increased from 15% (1970) to 56% (2008) and are projected to reach about 70% by 2030 (5). This increase has been observed in many parts of Africa at the rate of 23.2 per 100,000 (8). The highest incidence rates of 64.1 per 100,000 were in Southern Africa, followed by Northern Africa with 35.9 per 100,000 (3). Western Africa recorded 31.9 per 100,000, whereas Eastern and Western 23.9 per 100,000 and 13.2 per 100,000, respectively (3). Seychelles reported the highest increase among all African regions, 10.3%, during 2005–2018 (9). At the same time, Zimbabwe in earlier periods (1998–2002) reported the highest increase of 38.1%, which was eight times higher than those reported by the Gambia (4.7%) during 1997–1998 (10). Mali noted a steady increase of 6.7% during 1987–2017, with Malawi and Kenya 4.4 and 8.1%, respectively (9).

In South Africa, prostate cancer is the most common cancer among men across all provinces, with incidence rates of 61.8 per 100,000 (11), as reported by the National Cancer Registry (NCR). The incidence rates were 29.4 per 100,000 in 2007, with an almost double increase of 49.4 per 100,000 in 2017. However, it is acknowledged that these incidence rates might have been underestimated because NCR is a pathology-based cancer registry (11). Furthermore, not all geographic areas and sociodemographic groups have equal access to all tiers of health services (11). Similar findings from the population-based cancer registry in the Eastern Cape Province detail an increase of prostate cancer incidence from 4.1 (1998–2002) to 9.9 (2008–2012) per 100,000 (12, 13). A trend analysis of data from the African Cancer Registry Network (AFCRN) also shows an annual average percentage increase of 9.2% during 1998–2017, with the incidence rates ranging from 5.1 to 17.4 per 100,000 in this population (9).

A retrospective observational study is undertaken in the population living in the rural Eastern Cape Province using data from the Eastern Cape Cancer Registry (ECCR). This cancer registry covers nine magisterial areas and generates the high-quality standard data that has qualified its contribution to Cancer Incidence in Five Continents (CI5); Volumes X and XI. ECCR also participates in collaborative studies globally (CONCORD); (14) and regionally with the African Cancer Registry Network (AFCRN). Several studies on prostate cancer trends and incidence have been published internationally. However, in South Africa, there is a gap in published studies focusing on prostate cancer trends and incidence. Therefore, this study examines and describes prostate cancer trends in the Eastern Cape Province over 20 years, 1998–2017.

## Methods

This study used secondary data extracted from the ECCR database. The South Africa census of 2001 (15) and 2011 (16) was used to extrapolate population estimates. The average annual population at risk for the whole population was extrapolated from 2012 to 2017 by age, sex and, area using rates of change from the previously published estimates (13). Only patients reported in eight magisterial areas were included in this study. Patients from one urban magisterial area were excluded because this area was only included in the surveillance area since 2013. The analysis had 723 eligible prostate cancer (C61) patients.

## Data Analysis

For descriptive analysis, STATA version 14.0 was used. A direct standardization method was used to calculate age-specific and age-standardized rates (ASRs) per 100,000 population (17). This method was more appropriate for the analysis since it allows age-sex specific rates from each of the population under the study to be applied on a standard population, resulting standardized rates. The standard world population was used as the reference population (18). The following formula was used to calculate the age-specific rates; 10^5^ (d_n_/y_n_), where subscript n denotes the age group, d_n_ is the number of cases and y_n_ is the population at risk. The ASRs were calculated using a formula, where w_n_ is the world standard population (17, 18).


(1) $$\sum \left(\frac{d_{n}w_{n}}{y_{n}}\right)$$

Joinpoint Regression Program; version 4.5.0.1 (Statistical Research and Applications Branch, National Cancer Institute) was used to calculate APC from 1998 to 2017. APC measures rate changes over time and we used the Monte Carlo simulation of the permutation test, which is more restrictive to allow fewer Joinpoint than other models of the program. The standard error (SE) and 95% Confidence interval (CI) were premeditated using the adopted formula by Keyfitz (19) (SE = R/square root of N) where: R = (age-adjusted) rate and N = number of events (patients). The estimated SE was then used to compute the 95% confidence interval rates. The standard formula for determining the 95% CI was R ± (1.96 × SE) (19).

## Results

The patients' age ranges between 31 and 97 years, with a mean age of 64 years. The majority were black Africans (99.8%), Indian (0.1%), and colored (0.1%). Basis of diagnosis showed improvement in quality indicators, and the clinical diagnosis was 25.6% vs. the 37.2% of histology in 1998–2007, and histology verification improved to 65.8% in the 2008–2017 period (Table 1).

**Table 1:** Basis of diagnosis; 1998–2017.

**Basis of diagnosis**	**1998–2007**		**2008–2017**	
	***N*** **= 164**		***N*** **= 559**	
Clinical only	42	25.6%	39	7.0%
Laboratory test	61	37.2%	152	27.2%
Histology of primary	61	37.2%	368	65.8%
Total	164	100%	559	100%

Table 2 shows the ASR and APC with confidence intervals (CI) and the *P*-value for the entire study period. Variations in the incidence rates were observed across the magisterial areas for the whole period under observation; 1998–2017. Lusikisiki incidence rates increased significantly (*p* = 0.003) from ASR 2.6 to 53.4 per 100,000 population (95% CI: 0.8–4.4 to 95% CI: 45.5–61.4) with a significant annual increase of APC; 20.4. Incidence rates in other magisterial areas increased significantly with *p* ≤ 0.05. These included Centane with ASR of 5.5–21.7 per 100,000 population (95% CI: 2.3–8.8 to 95% CI: 15.8–27.6) with annual increase of APC; 9.8, Flagstaff with ASR of 1.3–14.1 per 100,000 population (95% CI: 0.0–3.1 to 95% CI: 8.4–19.9) with annual increase of APC; 12.7, Bizana with ASR 2.3–12.1 per 100,000 population (95% CI: 0.3–4.3 to 95% CI: 7.9–16.3) with annual increase of APC; 9.9, Nqamakwe with ASR 6.0–8.7 per 100,000 population (95% CI: 2.1–9.9 to 95% CI: 4.3–13.1) with annual increase of APC; 2.3. Conversely, in Butterworth, incidence rates decreased from ASR 15.2 to 11.5 per 100 000 population (95% CI: 8.6–21.9 to 95% CI: 6.2–16.7). The annual percentage change in Butterworth population was APC; −1.3.

**Table 2:** Age-standardized incidence rates and annual percentage change by magisterial areas.

**Periods**	**1998**–**2002**		**2003**–**2007**		**2008**–**20012**		**2013**–**2017**		
**Magisterial areas**	***N***	**ASR 95% CI**	***N***	**ASR 95% CI**	***N***	**ASR 95% CI**	***N***	**ASR 95% CI**	**APC (*P*-value)**
Butterworth	6	**15.2** (8.6–21.9)	9	**6.3** (2.2–10.5)	16	**10.7** (5.5–15.9)	18	**11.5** (6.2–16.7)	–**1.3** (0.760)
Bizana	5	**2.3** (0.3–4.3)	12	**5.3** (2.4–8.2)	23	**8.4** (4.9–12.0)	34	**12.1** (7.9–16.3)	**9.9** (0.035)^*^
Centane	11	**5.5** (2.3–8.8)	17	**9.3** (5.0–13.6)	22	**8.8** (5.0–12.5)	53	**21.7** (15.8–27.6)	**9.8** (0.046)^*^
Flagstaff	2	**1.3** (0.0–3.1)	6	**6.1** (1.2–10.9)	14	**7.1** (3.1–11.1)	23	**14.1** (8.4–19.9)	**12.7** (0.054)^*^
Idutywa	6	**3.6** (0.7–6.5)	4	**2.1** (0.0–4.2)	9	**4.9** (1.7–8.1)	13	**7.2** (3.3–11.1)	**6.1** (0.181)
Lusikisiki	8	**2.6** (0.8–4.4)	30	**9.6** (6.3–12.9)	74	**20.3** (15.6–25.1)	173	**53.4** (45.5–61.4)	**20.4** (0.003)^*^
Nqamakwe	9	**6.0** (2.1–9.9)	11	**7.4** (3.2–11.6)	13	**7.8** (3.4–12.2)	15	**8.7** (4.3–13.1)	**2.3** (0.038)^*^
Willowvale	8	**3.9** (1.2–6.5)	17	**9.0** (5.0–12.9)	21	**8.7** (4.9–12.5)	28	**10.9** (6.8–14.9)	**4.9** (1.950)
Overall Total	55	**4.5 (3.5–5.6)**	109	**8.5 (7.1–9.9)**	192	**10.6 (9.0–12.1)**	357	**21.4 (18.0–22.2)**	9.7 (0.001)^*^

* *P value was significant at ≤ 0.05*.

**Figure 1:**
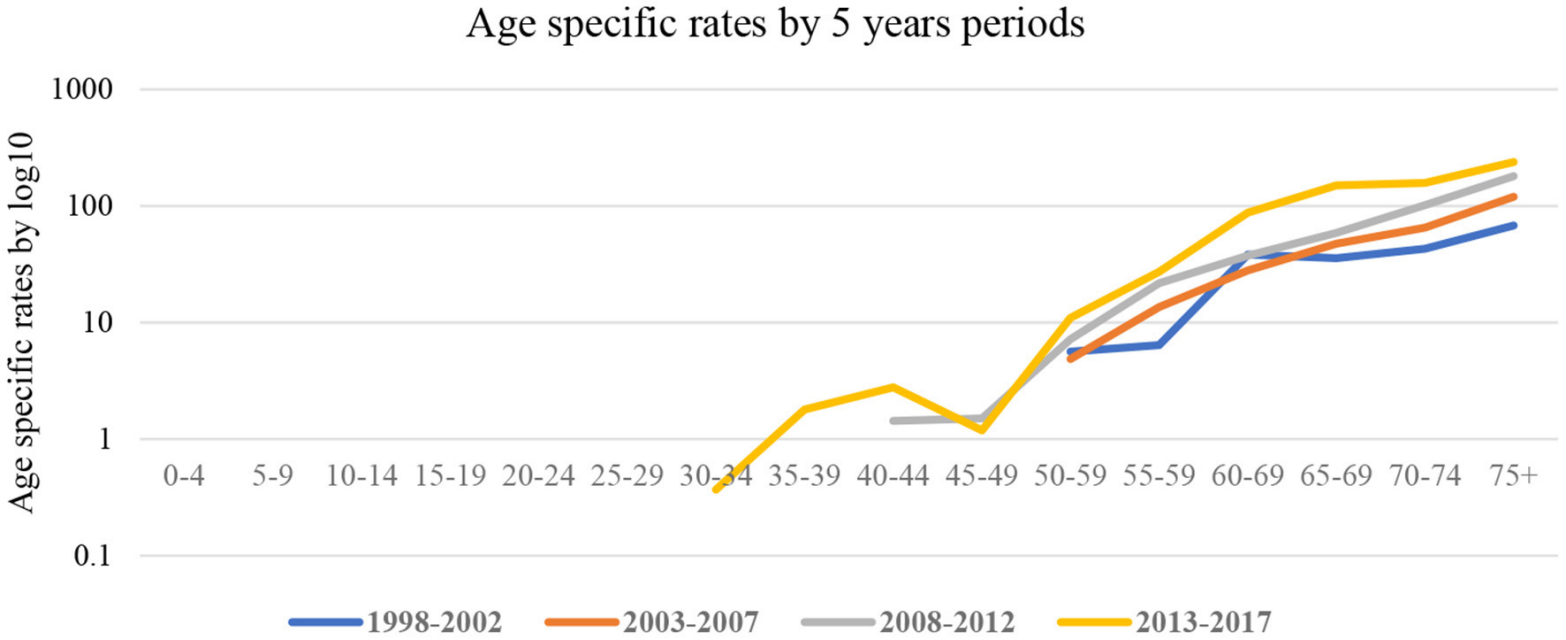
Age-specific rates in four periods; 1998–2002; 2003–2007; 2008–2012; 2013–2017.

Figure 1 shows age-specific rates calculated using the 5-year group from 1998 to 2017. During early observation periods, 1998–2002 and 2003–2007, the risk of cancer was amongst the age group 50–59 years and continued to age 75 years. However, during the 2008–2012 and 2013–2017 periods, a shift to younger age groups; 40–49 years was observed. ASRs increased from 7.2 per 100,000 population (1998–2002) to 68 per 100,000 (2008–2012) and 238 per 100,000 (2013–2017).

**Figure 2:**
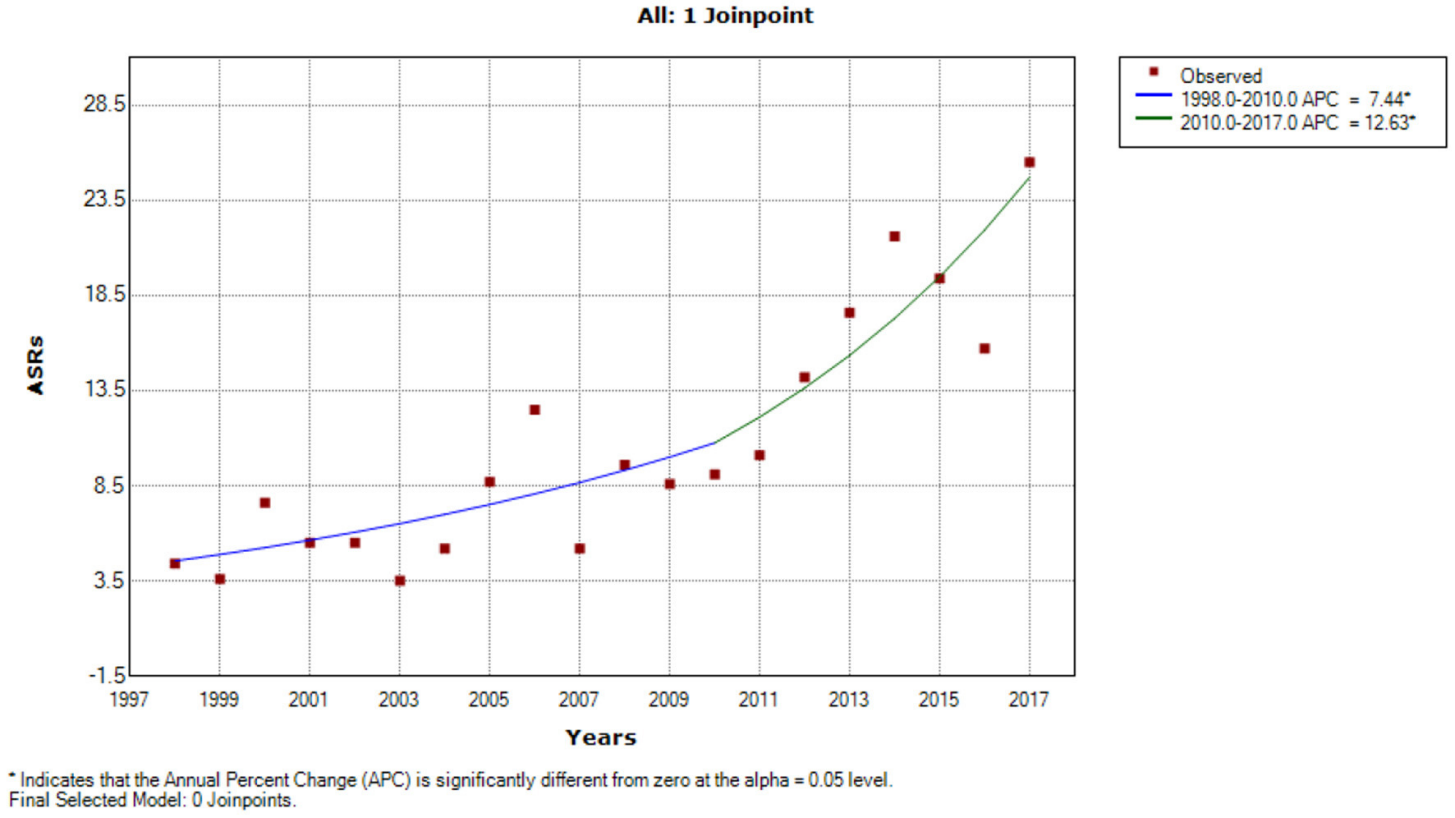
Prostate cancer risk annual percentage change over time; 1998–2017 period.

A Joinpoint regression model was applied to assess prostate trends over time. A statistically significant difference was observed (Figure 2). In the early observation period, incidence trends were low (ASR 3.9) until 2010, when a considerable increase was observed (ASR 12.0). From the beginning of the observation period, 1998–2010 APCs of 7.4% (95% CI = 1.0–14.3) was observed in 1998–2010 and increased to 12.6% (95% CI = 3.8–22.2) in 2010–2017.

## Discussion

Esophageal cancer has been significant for more than six decades in the population under observation (20–22). Scientists at the South African Medical Research Council (SAMRC) conducted several studies to understand the epidemiology of esophageal cancer. Subsequently, a unique cancer registry was established to monitor patterns and variations over time (20–22). However, since 1998, the unique cancer registry has developed and expanded to be population-based. Since the inception of the population-based cancer registry (PBCR), prostate cancer has been amongst the most common cancers in men but at significantly low rates, between ASR 4.1 (1998–2002) and ASR 9.9 (2008–2012) (12, 13). Histologically verified prostate cancer diagnoses improved significantly compared to the previous 10 years, from 37.2% (1998–2007) to 66.3% (2011–2017). Verified diagnoses improved the quality of data for patients reported and are another indicator of better disease management. There has been a significant increase in the overall number of patients observed from 55 (1998–2002) to 357 (2013–2017). The overall ASR increased from 4.1 per 100,000 (1998–2002) to 21.4 per 100,000 (2013–2017). ASRs were stable among young men (under 50 years) between 1998 and 2012. However, a significant increase among men under 50 in 2013–2017 was observed. Overall trends increased by 7.4–12.6% during 1998–2017. Our findings were consistent with a Zimbabwean study that found a 6.4% increase of prostate cancer incidence rates from 1991 to 2010 (23) and a 2.5-fold increase in Maputo (Mozambique) from 1956 to 2017 (24).

Trends differ by geographical area, were very high in the northern region (Bizana, Flagstaff, and Lusikisiki) compared to the southern region (Butterworth, Centane, Idutywa, Nqamakwe, and Willowvale) of the surveillance area. The prostate cancer trends constantly increased in Lusikisiki from 4.4 per 100,000 to 53.4 per 100,000 with an annual increase of 20.4%. In Bizana, Centane, Flagstaff, and Nqamakwe, incidence rates constantly increased over time; however, Idutywa and Willowvale remained constant. Conversely, incidence rates decreased in Butterworth from ASR 15.2 to 11.5 per 100,000, though not significantly with APC; −1.3.

This study observed the variation of prostate cancer incidence by magisterial area, with the most significant increase in Lusikisiki and the lowest in Idutywa. These findings compare with those observed in the systematic review by Dasgupta et al. (20). Dasgupta et al. argued that the cause of incidence variations observed are based on varying screening policies (20). Another similar study in the Eastern Cape Province reported a low incidence of prostate cancer and was associated with poor screening within the region (13). The NCR reported an increase in prostate cancer between 29.6 and 49.4 per 100,000 population over 2007–2017 (11).

In sub-Saharan Africa (SSA), increased prostate cancer incidence rates were reported, with APC that varies by 7-fold between the populations (9). Seychelles and Zimbabwe (Harare) recorded the highest ASR (21). The improved health system, including the recognition of screening using protein-specific antigen (PSA) tests, directly influenced this increase in SSA (21).

## Conclusion

For many decades, esophageal cancer was the most typical among men and public health concern in this area; however, this study shows a change in the cancer profile. The incidence of prostate cancer increased significantly in the Eastern Cape and hotspot were noted within the area. There is limited research, particularly in this area, and the cause of the increased incidence rates may be the results of the population aging, and other risk factors are unknown yet. We recommend that further investigations into these risk factors are imperative, including monitoring the hotspots. The case-control cohort study will be crucial to understand the risk factors associated with increase prostate cancer incidence in the area. Screening for early detection of the disease remains the critical factor that increases awareness of this disease and improves the quality of life and survival.

## Data Availability Statement

The raw data supporting the conclusions of this article will be made available by the authors, without undue reservation.

## Author Contributions

TR conceptualized, analyzed the data, compiled the results, and wrote the manuscript. AN and NS provided the data, conceptualized, and reviewed the manuscript. NIMS supervised the concept development and reviewed the manuscript.

## Conflict of Interest

The authors declare that the research was conducted in the absence of any commercial or financial relationships that could be construed as a potential conflict of interest.

## Publisher's Note

All claims expressed in this article are solely those of the authors and do not necessarily represent those of their affiliated organizations, or those of the publisher, the editors and the reviewers. Any product that may be evaluated in this article, or claim that may be made by its manufacturer, is not guaranteed or endorsed by the publisher.
